# Tetrahydro *iso*-Alpha Acids from Hops Improve Glucose Homeostasis and Reduce Body Weight Gain and Metabolic Endotoxemia in High-Fat Diet-Fed Mice

**DOI:** 10.1371/journal.pone.0033858

**Published:** 2012-03-28

**Authors:** Amandine Everard, Lucie Geurts, Marie Van Roye, Nathalie M. Delzenne, Patrice D. Cani

**Affiliations:** Université catholique de Louvain, Louvain Drug Research Institute, Metabolism and Nutrition Research Group, Brussels, Belgium; Paris Institute of Technology for Life, Food and Environmental Sciences, France

## Abstract

Obesity and related metabolic disorders such as insulin resistance and type 2 diabetes are associated with a low-grade inflammatory state possibly through changes in gut microbiota composition and the development of higher plasma lipopolysaccharide (LPS) levels, i.e. metabolic endotoxemia. Various phytochemical compounds have been investigated as potential tools to regulate these metabolic features. *Humulus lupulus* L. (hops) contains several classes of compounds with anti-inflammatory potential. Recent evidence suggests that hops-derived compounds positively impact adipocyte metabolism and glucose tolerance in obese and diabetic rodents via undefined mechanisms. In this study, we found that administration of tetrahydro *iso*-alpha acids (termed META060) to high-fat diet (HFD)-fed obese and diabetic mice for 8 weeks reduced body weight gain, the development of fat mass, glucose intolerance, and fasted hyperinsulinemia, and normalized insulin sensitivity markers. This was associated with reduced portal plasma LPS levels, gut permeability, and higher intestinal tight junction proteins Zonula occludens-1 and occludin. Moreover, META060 treatment increased the plasma level of the anti-inflammatory cytokine interleukin-10 and decreased the plasma level of the pro-inflammatory cytokine granulocyte colony-stimulating factor. In conclusion, this research allows us to decipher a novel mechanism contributing to the positive effects of META060 treatment, and supports the need to investigate such compounds in obese and type 2 diabetic patients.

## Introduction

Obesity is characterized by a massive expansion of the adipose tissue [1]. Growing evidence suggests that obesity and related metabolic disorders such as insulin resistance, type 2 diabetes, and cardiovascular diseases are associated with a low-grade inflammatory state [2]. Recently, the origin of this inflammatory tone has been linked to the development of higher plasma lipopolysaccharide (LPS) levels, or metabolic endotoxemia [3], [4]. LPS is a cell-wall component of Gram-negative bacteria considered as the most potent inducer of inflammation. We and others have demonstrated a tight relationship between metabolic endotoxemia, changes in gut microbiota composition, the development of glucose intolerance, insulin resistance and type 2 diabetes in both rodents and humans [3]–[13].

Numerous phytochemical compounds have been studied as potential tools to regulate glucose homeostasis, adipose tissue development and inflammatory tone. Among these is the widely studied plant genus *Humulus* (belonging to the family of Cannabaceae) [14]. The species *Humulus lupulus* L. (hops), is a plant that has been used for medicinal purposes for centuries and contains several classes of compounds with anti-inflammatory potential [14]–[16]. Growing evidence suggests that tetrahydro *iso*-alpha acids (termed META060) derived from hops inhibited LPS–stimulated prostaglandin E2 (PGE_2_) production, nitric oxide formation, cyclooxygenase 2 (COX-2) abundance, and the pro-inflammatory transcription factors nuclear factor kappa B (NF-κB) pathway in macrophages [15]. Moreover, these compounds exert anti-inflammatory activity on LPS-stimulated tumor necrosis factor alpha (TNF-α) and interleukin-6 (IL-6) production in isolated peripheral blood mononuclear cells [16]. In addition to these effects hops-derived compounds (including *iso*-alpha acids) have been found to block TNF-α-induced production of IL-6 and to inhibit the transactivation of NF-κB, activator protein-1 (AP-1), and cAMP-response element-binding protein (CREB) in fibroblasts [17]. More recently, it has been proposed that hops has positive impact on adipocyte metabolism [18] and glucose tolerance in obese and diabetic rodents; however, the exact mechanisms explaining these effects are not well characterized [19], [20]. Therefore, in the present study we investigated the impact of a well-defined hop extract (META060) on body weight gain, fat mass development, glucose homeostasis and gut barrier function markers in diet-induced obese and type 2 diabetic mice.

## Materials and Methods

### Materials

META060 was supplied by Metagenics, Inc. (Gig Harbor, WA, USA); the chemical composition of META060 has been described previously [15], [16]. C57BL/6J mice were obtained from Charles River Laboratories International, Inc. (Brussels, Belgium).

### Mice

A set of 10-week-old C57BL/6J mice (30 mice, n = 10/group) were housed in groups of three or four mice/cage, with free access to food and water. Mice were fed a control diet (CT) consisted of normal chow (Research diet, New Brunswick, NJ, USA), a high-fat diet (HFD) (D12492, Research diet) containing 45% fat (kcal/100 g) and 20% carbohydrates (kcal/100 g), or a high-fat diet supplemented with 0.1% META060 (HFD-META060). Treatment continued for 8 weeks. Food intake was recorded as a cumulative intake over a week and once a week during the treatment period.

This study was performed in strict accordance with the guidelines of the local ethics committee (ethical committee of the Université catholique de Louvain for animal experiments specifically approved this study that received the agreement n° 2010/UCL/MD/022) and are in accordance with the European recommendation 2007/526/CE, translated in the Belgian Law of April 6, 2010, regarding the protection of laboratory animals. The laboratory has received the agreement number LA1230314 from the Ministry of Agriculture. All the animals are housed in cages with enrichment in a controlled light/dark cycle, temperature and humidity environment. Every effort was made to minimize suffering during manipulations and oral gavage. Food was removed 2 h after the onset of the daylight cycle and mice were fasted 6-h as previously described [3], [4], then all the animals have been anesthetized with isoflurane (Forene®, Abbott, Queenborough, Kent, England) before exsanguination and tissue sampling, then mice were killed by cervical dislocation.

### Oral glucose tolerance test

Oral glucose tolerance tests were performed after 7 weeks of treatment. Food was removed 2 h after the onset of the daylight cycle and mice were treated after a 6-h fasting period as previously described [3], [4].

### Tissue sampling

Mice were anesthetized with isoflurane (Forene®, Abbott, Queenborough, Kent, England) after a 6-h fasting period. Blood samples and tissues were harvested for further analysis. Epididymal, subcutaneous and visceral adipose deposits were precisely dissected and weighed. The jejunum segments and adipose tissues were immediately immersed in liquid nitrogen and stored at −80°C for further analysis.

### Biochemical analyses

Portal plasma LPS concentration was measured by using Endosafe-MCS (Charles River Laboratories, Lyon, France) based on the Limulus amaebocyte Lysate (LAL) kinetic chromogenic methodology that measures color intensity directly related to the endotoxin concentration in a sample as previously described [5]. Serum were diluted 1/10 with endotoxin free buffer to minimize interferences in the reaction (inhibition or enhancement) and heated 15 min at 70°C. Each sample was diluted 1/70 or 1/100 with endotoxin-free LAL reagent water (Charles River Laboratories) and treated in duplicate and two spikes for each sample were included in the determination. All samples have been validated for the recovery and the coefficient variation. The lower limit of detection was 0.005 EU/ml.

Plasma insulin concentration was determined in 25 µl of plasma using an ELISA kit (Mercodia, Upssala, Sweden) as previously described [5], 21.

Plasma IL-10 and granulocyte colony-stimulating factor (G-CSF) were determined in duplicate by using a Bio-Plex Pro Assays kit (Bio-Rad, Nazareth, Belgium) and measured by using Luminex (Bio-Rad Bioplex, Bio Rad) following the manufacturer's instructions.

Intestinal phosphatase alkaline (IAP) activity was measured in 30 mg of jejunum tissue following the kinetic chromogenic methodology that measures color intensity directly related to the kinetic hydrolysis (30 min) of the *p*-nitrophenylphosphate (pNNP) in *p*-nitrophenol (pNP) as previously described [22].

### Occludin immunoblot analysis

Samples (30 mg) were homogenized with TissueLyser II (Qiagen) and lysed in buffer (100 mM KCl, 3 mM NaCl, 3.5 mM MgCl2, and 10 mM HEPES; pH 7.4) containing 1% Triton X-100, 1% protease inhibitor cocktail (P8340, Sigma-Aldrich, Saint Louis, USA), and phosphatase inhibitors (100 mM NaF and 200 mM Na3VO4) and incubated 30 min at 4°C. After centrifugation (15000 g for 15 min at 4°C) the pellet was dissolved in SDS buffer (4% (wt/vol) SDS, 0.75 M Tris (pH 8.8), 15% glycerol, and 20 mM DTT) and heated 10 min at 65°C thereby corresponding to the insoluble fraction. The protein concentration was measured by the Bradford method. Proteins (60 µg/lane) were fractionated on a 8% SDS-PAGE gel and transferred to nitrocellulose membranes (Hybond-ECL, Amersham). Membranes were blocked in 0,1% Tween 20 in TBS with 5% (wt/vol) nonfat dry milk for 2 h at room temperature and then probed with rabbit anti-Occludin (Invitrogen) (0,5 µg/ml) antibodies overnight at 4°C or with mouse anti-beta actin (Abcam) (1/10000) for 1 hour. After washing, membranes are incubated for 1 h with HRP-conjugated secondary antibodies (goat anti-rabbit 1∶10,000, Chemicon or rabbit anti-mouse 1∶2500, Dako). Immunocomplexes were visualized using ECL Western blotting detection reagents (SuperSignal West Pico Substrate, Thermo Scientific).

### RNA preparation and real-time qPCR analysis

Total RNA was prepared from tissues using TriPure reagent (Roche). Quantification and integrity analysis of total RNA was performed by running 1 µl of each sample on an Agilent 2100 Bioanalyzer (Agilent RNA 6000 Nano Kit, Agilent). cDNA was prepared by reverse transcription of 1 µg total RNA using a Reverse Transcription System kit (Promega, Leiden, The Netherlands). Real-time PCRs were performed with the StepOnePlus™ real-time PCR system and software (Applied Biosystems, Den Ijssel, The Netherlands) using Mesa Fast qPCR™ (Eurogentec, Seraing, Belgium) for detection according to the manufacturer's instructions. RPL19 RNA was chosen as the housekeeping gene. Primer sequences for RPL-19, ZO-1 and Occludin were previously described [7]. All samples were run in duplicate in a single 96-well reaction plate, and data were analyzed according to the 2^−ΔCT^ method. The identity and purity of the amplified product was checked through analysis of the melting curve carried out at the end of amplification.

### Statistical analyses

The data are expressed as the mean ± SEM. Differences between groups were assessed using one-way ANOVA, followed by *post-hoc* Tukey-tests or two-tailed Student's t-test. Data were analyzed using GraphPad Prism version 5.00 for Windows (GraphPad Software, San Diego, CA, USA). The results were considered statistically significant at P<0.05.

## Results

### META060 reduces high-fat diet-induced body weight gain and fat mass development

**Figure 1:**
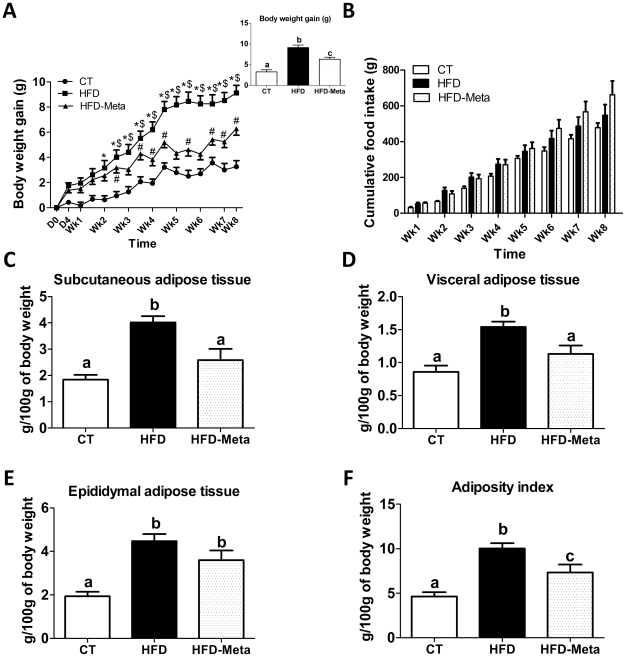
META060 reduces high-fat diet-induced body weight gain and fat mass development. (**A**) Body weight gain (g) evolution, *inset* correspond to total body weight gain, (**B**) cumulative food intake (g), (**C**) subcutaneous, (**D**) visceral, (**E**) epididymal adipose tissues weight, and (**F**) adiposity index (corresponding to the sum of the three adipose depots) expressed as the percentage of total body weight of mice fed a control diet (CT), high-fat diet (HFD) or high-fat diet supplemented with 0.1% META060 (HFD-META060). n = 10 mice/group. Data are expressed as mean ± SEM. Different superscript letters were significantly different (P<0.05) according to one-way ANOVA. Interaction Treatment×Time P<0.0001; Treatment P<0.0001; Time P<0.0001. *P<0.05 HFD versus CT, $P<0.05 HFD-Meta versus HFD, #P<0.05 HFD-Meta versus CT according to Two-way ANOVA.

We found that mice fed with a high-fat diet (HFD mice) significantly and progressively gained weight throughout the study as compared to control (CT) mice (Figure 1A and inset, Figure S1). Interestingly, HFD-META060 mice exhibited significant reduced body weight gain than HFD mice (Figure 1A), whereas food intake was not affected by the treatment (Figure 1B). Similarly, both subcutaneous and visceral adipose depots were significantly lower in HFD-META060 mice than in HFD mice (Figure 1C and D), whereas a trend toward a decrease was found for the epididymal fat tissue that remained significantly higher in HFD-META060 than in the CT mice (Figure 1E). Overall, META060 significantly decreased the total adiposity as compared to the HFD-fed mice (Figure 1F), although this parameter was not fully returned to the levels seen in CT mice.

### META060 improves glucose tolerance in obese and type 2 diabetic mice

**Figure 2:**
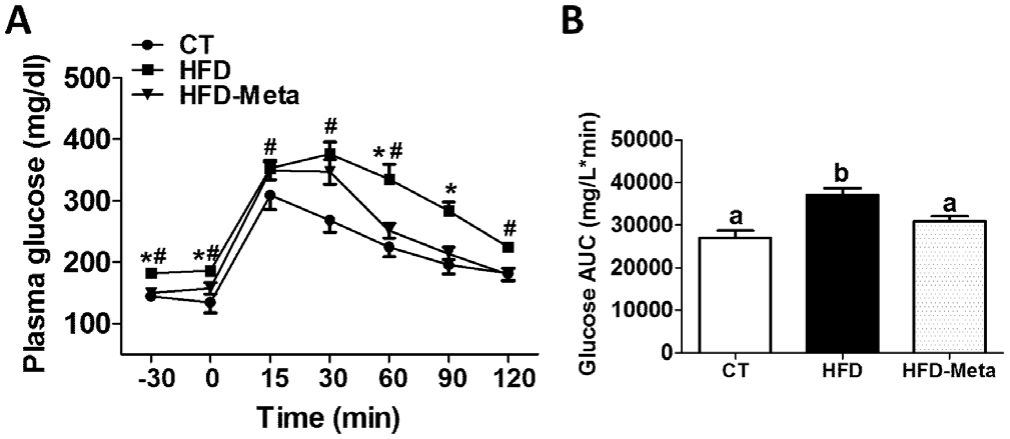
META060 improves glucose tolerance in obese and type 2 diabetic mice. (**A**) Plasma glucose levels and (**B**) area under the curve following 2 g/kg glucose oral challenge measured in freely moving mice fed a CT, HFD or HFD-META060 diet. Data are mean ± SEM. n = 10 mice/group. *P<0.05 HFD versus HFD-META060, ^#^P<0.05 HFD versus CT diet determined by a two-tailed Student's t-test. Data with different superscript letters were significantly different (P<0.05) according to one-way ANOVA.

HFD feeding promoted type 2 diabetes and glucose intolerance as shown by the significantly higher plasma glucose levels in the fasting state and following an oral glucose load (Figure 2A). HFD-META060 feeding improved glucose homeostasis as shown by the normalized fasting glycemia and the improved glucose tolerance (Figure 2A). In addition, area under the curves (AUC) measured during 2 h following the oral glucose challenge was equivalent between CT mice and HFD-META060 mice, whereas AUC in HFD mice was significantly increased compared to the other 2 groups (Figure 2B).

### META060 protects against high-fat diet-induced insulin resistance and fasted hyperinsulinemia in obese and type 2 diabetic mice

**Figure 3:**
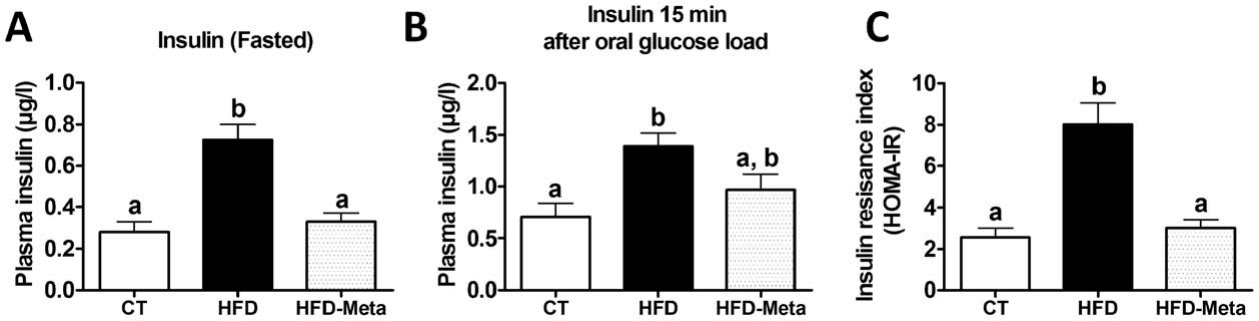
META060 protects against high-fat diet-induced insulin resistance and fasting hyperinsulinemia in obese and type 2 diabetic mice. (**A**) Fasted plasma insulin levels, (**B**) glucose-induced insulin secretion (15 min after an oral glucose load), and (**C**) insulin resistance index (Homeostasis model assessment of insulin resistance (HOMA-IR) measured in mice fed a CT, HFD or HFD-META060 diet. Data are mean ± SEM. n = 10 mice/group. Data with different superscript letters were significantly different (P<0.05) according to one-way ANOVA.

Fasting hyperinsulinemia and insulin resistance are both hallmark of obesity and type 2 diabetes. Here, we found that HFD-META060 mice were completely resistant to the high-fat diet-induced hyperinsulinemia in both fasting and following oral glucose load (Figure 3A and B). More importantly, HFD mice exhibited a 4-fold increase in the insulin resistance index (HOMA-IR), whereas HFD-META060 and CT mice were resistant to the development of insulin resistance, as shown by the similar HOMA-IR scores (Figure 3C).

### META060 modulates plasma cytokines in obese and type 2 diabetic mice

Given that IL-10 has been found to protect against diet-induced insulin resistance [23] and to be positively associated with insulin sensitivity [24], we measured this cytokine in the plasma. We found that in HFD-META060 mice plasma IL-10 (pg/ml) increased by about 46% (CT 80.6±7.5, HFD 72.7±3.9, HFD-META060 106.3±13.2, HFD vs HFD-META060: *P* = 0.07). We next assayed plasma levels of the cytokine G-CSF, which has been considered as a key pro-inflammatory regulator in diet-induced obese mice [25], [26]. We observed that HFD feeding increased G-CSF levels (pg/ml) by about two-fold as compared to CT mice (*P* = 0.06), whereas HFD-META060 mice exhibited normalized levels (CT 53±5.1, HFD 105.7±28, HFD-META060 50.6±3.6, HFD vs HFD-META060: *P* = 0.055).

### META060 reduces high-fat diet-induced metabolic endotoxemia in obese and type 2 diabetic mice

**Figure 4:**
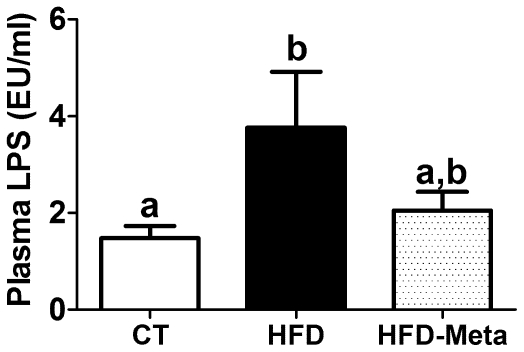
META060 reduces high-fat diet-induced metabolic endotoxemia in obese and type 2 diabetic mice. (**A**) Portal plasma lipopolysaccharide (LPS) levels measured in mice fed a CT, HFD or HFD-META060 diet. Data are mean ± SEM. n = 10 mice/group. Data with different superscript letters were significantly different (P<0.05) according to one-way ANOVA.

We previously found that metabolic endotoxemia (increased plasma LPS levels) contributed to the alteration of glucose tolerance and triggered insulin resistance [3]. In addition, plasma LPS correlated with plasma glucose, fasting insulinemia and predicted type 2 diabetes incident [6], [13], [27], [28]. Therefore, we measured the impact of META060 on high-fat diet induced metabolic endotoxemia. We found that HFD feeding significantly increased plasma LPS levels, and HFD-META060 fed mice were resistant to the HFD induced metabolic endotoxemia (Figure 4).

### META060 tends to increase intestinal phosphatase alkaline (IAP) activity

Among the mechanisms involved in decreased plasma LPS, several studies have demonstrated that IAP can be considered as a protective factor involved not only in gut barrier integrity but also in LPS detoxification [10], [29]–[31]. We found that HFD-META060 group increase IAP activity by about 25% as compared to HFD group (CT 14.45±1.34, HFD 13.98±0.69, HFD-META060 17.18±1.42 nmoles of hydrolyzed substrates/min per mg of proteins, HFD vs HFD-META060: *P* = 0.07).

### META060 reduces gut permeability via occludin and ZO-1 tight-junction proteins modulation in high-fat diet-induced obese and type 2 diabetic mice

**Figure 5:**
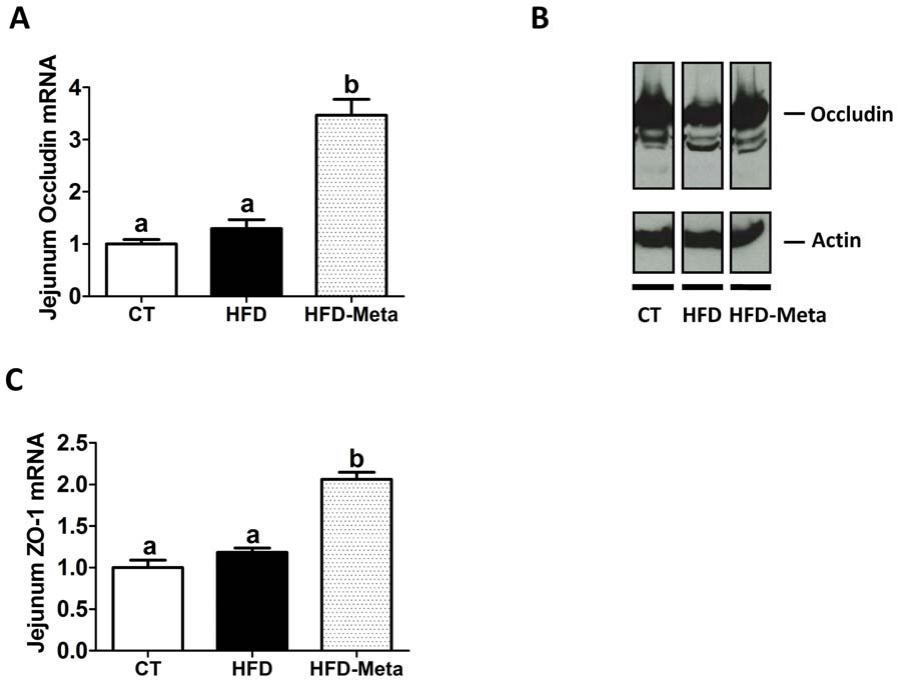
META060 increases occludin, ZO-1 tight-junction proteins mRNA expression and improves Occludin protein distribution in high-fat diet-induced obese and type 2 diabetic mice. (**A**) Zonula occludens (ZO-1) and (**C**) occludin mRNA expression levels, (**B**) representative blot of Occludin protein expression from insoluble cellular fraction in the jejunum of mice fed a CT, HFD or HFD-META060 diet. Actin was used as loading control (n = 5 mice/group). mRNA data are mean ± SEM. n = 10 mice/group. Data with different superscript letters were significantly different (P<0.05) according to one-way ANOVA.

In addition to the IAP activity, we and others have previously shown that gut barrier function controls plasma LPS levels in both diet-induced and genetically obese and type 2 diabetic mice, possibly through the up-regulation of two key tight junctions proteins, zonula occludens-1 (ZO-1) and occludin [4], [7], [8], [10], [11]. Here we found that HFD-META060 mice displayed a 2- to 3-fold increase in ZO-1 and occludin mRNA levels, respectively (Figure 5A and C). Tight junctions are structurally characterized as soluble or insoluble proteins by their partition into Triton X-100 buffer [11]. As shown on figure 5B, Occludin expression is lower in the insoluble protein fractions of HFD mice than HFD-META060, and control mice. These findings strongly suggest a shift of intestinal junctional protein from the cytoskeleton, causing a decrease in paracellular sealing, and thereby higher gut permeability in HFD mice.

Together with the plasma LPS levels, these data indicate that META060 feeding has positive impact on the gut barrier function.

## Discussion

In the present study, we characterized the impact of *Humulus lupulus* L.-derived compounds (i.e. META060) on several metabolic features that are associated with high-fat diet feeding. First, META060 treatment significantly reduced body weight gain and fat mass development following 8 weeks of high-fat diet. Second, META060 protected mice against diet-induced fasting hyperglycemia and hyperinsulinemia. Third, these effects were associated with improved glucose tolerance and normalized insulin sensitivity. In addition, we discovered the improvement of high-fat diet-induced metabolic endotoxemia to be a novel mechanism contributing to META060's metabolic effects. Given that these markers are all hallmarks of type 2 diabetes, these findings strongly support the role of META060 in the prevention of this disease.

Mice fed with hops-derived META060 exhibited resistance to high-fat diet-induced body weight gain and fat mass development. Given the fact that food intake was not different between HFD and HFD-META060 groups, it is likely that META060 contributed to the reduced fat mass development and thereby preventing body weight gain. It has been recently proposed that hops *iso*-alpha acids, structurally similar to META060, increase liver fatty acid oxidation and normalize adipocyte hypertrophy via the co-activation of PPARα and PPARγ [19], [20]. META060 (tetrahydro *iso*-alpha acids) may also possess such biological activity and this warrants future studies.

However, even though we observed lower body weight and fat mass in HFD-META060 mice, the adiposity index remained significantly higher than in normal chow fed mice. Nevertheless, fasting plasma glucose levels were completely normalized and, in accordance with this effect, glycemia returned to the basal level 2 hours after the oral glucose challenge, similar to what we observed in the control mice. Importantly, HFD-META060 mice were completely resistant to the diet-induced hyeprinsulinemia. This effect was corroborated by the absence of insulin resistance in HFD-META060 mice compared to the HFD mice which developed a marked insulin resistance. Therefore, these results strongly suggest that tetrahydro *iso*-alpha acids exert beneficial effect on plasma glucose, inflammation and insulin resistance independently of body weight loss or lower body fat accumulation. In accordance with this hypothesis, it has been previously demonstrated that these compounds inhibit LPS–stimulated PGE_2_, TNF-α, IL-6, nitric oxide and COX-2 abundance, as well as NF-κB pathway [15]–[17]. It is worth noting that previous study has shown that mice treated for 21 days with Meta060 did not exhibited any changes in body weight gain and food intake [32], further supporting that these compounds are not acting through food intake and or body weight-dependent mechanisms.

For instance, the study of VO2 and VCO2 by indirect calorimetry at rest and during physical activity could constitute one interesting perspective to investigate further.

Previous studies have shown in diet-induced and genetic obesity models that low-grade inflammatory tone causes insulin resistance [1], [33] which favors hyperinsulinemia and excessive hepatic and adipose tissue lipid storage [34], [35]. Extensive research has been dedicated to unravel the effects of inflammatory reactions on energy metabolism; however, the triggering factors linking high-fat diet-induced metabolic syndrome are not fully determined. Accumulating evidence suggests that various hops-derived compounds abolished LPS-induced inflammation via several mechanisms including NF-κB-dependent pathways [15]–[18], [32]. In the present study, we believe this important property contributes to the improved phenotype observed in HFD-META060 mice.

Plasma levels of the anti-inflammatory cytokine IL-10 have been found to positively correlate with insulin sensitivity [24] and IL-10 treatment protects against diet-induced insulin resistance [23]. In the present study, META060 treatment increased plasma IL-10 by about 46%. Although this increase was not statistically significant, we believe that it contributed to the improved phenotype observed. We also measured plasma levels of the pro-inflammatory cytokine G-CSF. G-CSF is known to exacerbate underlying inflammatory diseases [26] and increases in diet-induced obese mice [25]. We found a 2-fold decrease in G-CSF plasma levels in HFD-META060 mice compared to HFD mice.

In addition to these effects on the inflammatory tone, we identified that META060 feeding abolished high-fat diet-induced metabolic endotoxemia. Growing evidence shows that obesity and type 2 diabetes are associated with an altered gut microbiota composition and higher plasma LPS levels, although the underlying mechanisms are not fully understood. However, it is worth mentioning that obesity has been found to be associated with altered gut barrier function [4], [7], [8], [10], [11]. The maintenance on the gut barrier function is dependent of the tight junction proteins complex including two key markers ZO-1 and Occludin [36], [37]. Furthermore, several studies have shown that the increased plasma LPS levels and gut permeability are explained in part by the alteration of the expression of genes coding for tight junction proteins (ZO-1 and occludin) [4], [7], [8], [11]. In the present study, both ZO-1 and occludin mRNA expressions were significantly up-regulated following META060 treatment. In addition to the mRNA expression, we investigated Occludin protein distribution through its solubility profile. HFD mice exhibited decreased Occludin expression in the insoluble fraction, thereby suggesting that Occludin is delocalized from the junction protein complex associated with the cytoskeleton [11], [38] leading to altered gut barrier function. Therefore, we postulate that the increased Occludin protein levels found in the insoluble fraction contribute to the improved gut barrier function, hence unraveling the mechanism involved in the reduced metabolic endotoxemia and inflammation observed in HFD-META060 treated mice. In addition to these findings, we demonstrated that META060 treatment increases intestinal alkaline phosphatase activity by about 25%. Although, this effect did not reach significance, we may not rule out that such increased activity throughout the entire gastrointestinal tract not only contributes to reinforcing the gut barrier but might also be involved in LPS detoxification [30].

Therefore, we hypothesized that the improved metabolic endotoxemia could be due to several effects of META060 on gut barrier. Several lines of evidence are supporting this hypothesis. For instance, tight junction complexes are regulated through several canonical pathways such as the mitogen-activated protein (MAP) kinase and NF-κB pathways [39], [40], and META060 has been shown to interact with these pathways [15], [16]. Moreover, LPS has been proposed as a factor involved in the development of gut barrier disruption *per se* [41], [42], and hops *iso*-alpha acids and META060 have been shown to abolish LPS-induced inflammation and NF-κB transcription [15]–[17], [32]

In conclusion, META060 improves diet-induced obesity, fat mass development and associated metabolic disorders (glucose intolerance, insulin resistance). More importantly, META060 protects mice against high-fat diet-induced metabolic endotoxemia, a result associated with improved gut barrier markers (Occludin distribution and IAP) and lower inflammatory tone. This research allows us to decipher a novel mechanism contributing to the positive effects of tetrahydro *iso*-alpha acids treatment. Altogether, these data support the need to test this compound in obese and type 2 diabetic patients.

## Supporting Information

Figure S1 **META060 reduces high-fat diet-induced body weight gain.** Body weight evolution (g) of mice fed a control diet (CT), high-fat diet (HFD) or high-fat diet supplemented with 0.1% META060 (HFD-META060). n = 10 mice/group. Data are expressed as mean ± SEM.
(TIF)
